# Detection of Six Different Human Enteric Viruses Contaminating Environmental Water in Chiang Mai, Thailand

**DOI:** 10.1128/spectrum.03512-22

**Published:** 2022-12-19

**Authors:** Kattareeya Kumthip, Pattara Khamrin, Hiroshi Ushijima, Niwat Maneekarn

**Affiliations:** a Department of Microbiology, Faculty of Medicine, Chiang Mai University, Chiang Mai, Thailand; b Center of Excellence (Emerging and Re-emerging Diarrheal Viruses), Chiang Mai University, Chiang Mai, Thailand; c Department of Developmental Medical Sciences, The University of Tokyo, School of International Health, Graduate School of Medicine, Tokyo, Japan; d Department of Pathology and Microbiology, Nihon University School of Medicine, Tokyo, Japan; Wright State University

**Keywords:** enteric viruses, environmental water, Thailand, wastewater

## Abstract

A 2-year surveillance study into enteric viruses contaminating environmental water samples was conducted in the city of Chang Mai, Thailand. The aim of the study was to investigate the prevalence of six different human enteric viruses, specifically, adenovirus (AdV), astrovirus (AstV), enteroviruses (EVs), human parechovirus (HPeV), rotavirus (RV), and saffold virus (SAFV), contaminating several types of environmental water using PCR and reverse transcription-PCR (RT-PCR) methods. All targeted viruses were detected with different levels of prevalence. The levels ranged from 0.8 to 4.8% (AdV, 0.8%; AstV, 4.8%; EV, 0.8%; HPeV, 3.2%; RV, 0.8%; SAFV, 3.2%). A wide variety of human enteric virus genotypes, including AdV-41, AstV-MLB1, coxsackievirus A, HPeV1, 5, and 6, RV G4[P8], and SAFV-2 and 3 were detected. The overall picture of the 13 human enteric viruses that were detected in environmental water in Chiang Mai, Thailand, is also summarized in this study. The data and the findings of this study will provide a better understanding of the viral dynamics in environmental water. The detection of these viruses in environmental water indicates there is the potential for human infection from this source.

**IMPORTANCE** Human enteric viruses are a major cause of gastrointestinal illness, and these viruses can be introduced into environmental water through various routes. Viral contamination in water could play a significant role in human health. This study demonstrated the prevalence of six different enteric viruses, adenovirus, astrovirus, enteroviruses, human parechovirus, rotavirus, and saffold virus, contaminating environmental water. We also analyzed the overall prevalence of other enteric viruses that were in this area, and the findings revealed a wide diversity of the enteric viruses contaminating environmental water. The data provide a better understanding of the epidemiologic importance of viral contamination of the water and highlight the need for better management of wastewater disposal and effective environmental water treatment to prevent the human population from infection.

## INTRODUCTION

Human enteric viruses are a major cause of gastrointestinal illness and can be introduced into environmental water through various routes (1, 2). The majority of enteric viruses are transmitted by the fecal-oral route and remain highly stable in an environment for a long period of time (3). The viruses continue to be shed in the feces of infected individuals, contaminating the water sources, persisting in the water for several days to several weeks (3). Due to this, enteric viruses play an important role in water-associated gastroenteritis outbreaks and sporadic cases, creating a circle of infection. Infection by human enteric viruses is often asymptomatic in healthy people but may cause clinical symptoms ranging from mild diarrhea to chronic or severe symptoms in young children, the elderly, and immunocompromised individuals (4).

Contamination by several human enteric viruses in environmental water has been described (2). Viruses of primary concern for waterborne disease outbreaks include noroviruses, hepatitis A virus, hepatitis E virus, adenovirus, astrovirus, enteroviruses, and rotavirus. Our previous studies have detected a number of gastroenteritis viruses in river water and wastewater, including Aichivirus, bufavirus, human bocavirus, noroviruses and sapovirus, picobirnavirus, and salivirus (5–10). In order to get more comprehensive information and an overall picture of the contamination of environmental water by enteric viruses, this study aimed to continue investigating the occurrence of adenovirus, astrovirus, enteroviruses, human parechovirus, rotavirus, and saffold virus in samples from key areas reflecting a variety of environmental water sites. Surveillance of a panel of enteric viruses in environmental water will provide a better understanding of the impact of waterborne viruses on public health and facilitate a risk assessment of water-related human infection.

## RESULTS

### Prevalence of six enteric viruses detected in environmental water samples.

**FIG 1:**
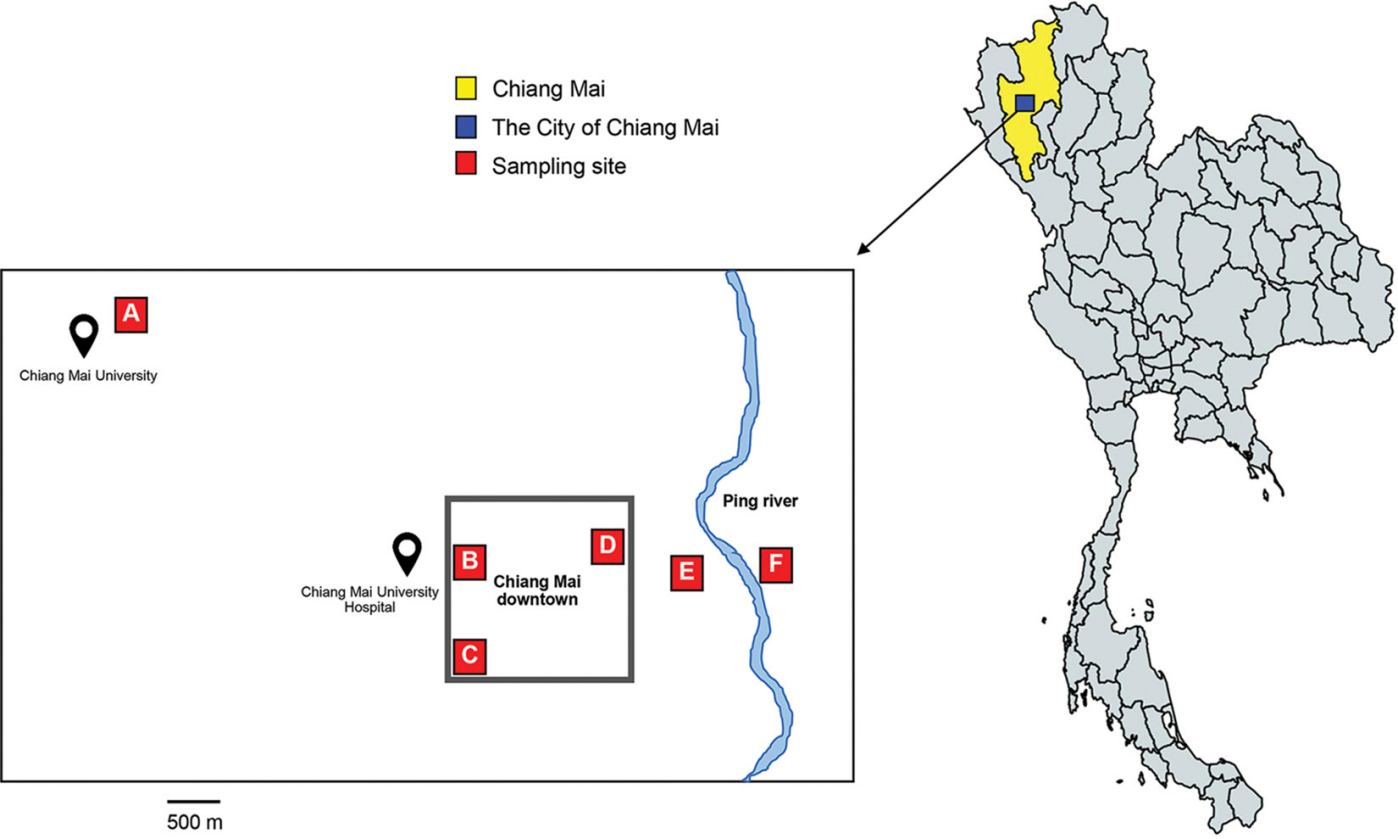
(A to F) Sampling sites at six different locations in Chiang Mai, Thailand: Ang Kaew reservoir (A), Suandok Canal (B), Buak Hard Garden Reservoir (C), Sompech Canal (D), Mae Kha Canal (E), and Ping River (F). Map was modified from mapchart.net.

Of the 126 water samples collected from six different sampling sites (Fig. 1), 17 samples were positive for enteric viruses (13.5%). Six different enteric viruses were detected in environmental water, with astrovirus being the predominant virus (4.8%, 6 out of 126), followed by human parechovirus and saffold virus (each at 3.2%, 4 out of 126). Adenovirus, enteroviruses, and rotavirus were detected at a low prevalence (each at 0.8%, 1 out of 126) (Table 1). Among the six different water sampling locations, Suandok and Sompech Canals were irrigation water, Ang Kaew and Buak Hard were reservoir water, Ping River was river water, and the Mae Kha Canal was wastewater. It was found that the Mae Kha Canal was the most polluted sampling site, containing all targeted viruses except for rotavirus. Of the 17 positive samples, 14 (82.4%) were collected from the Mae Kha Canal, whereas none of the targeted viruses were detected in water samples from Ang Kaew Reservoir or Sompech Canal. One targeted virus was detected in water samples from Suandok Canal, Ping River, and Buak Hard Reservoir.

**Table 1:** Prevalence of six enteric viruses detected in environmental water samples collected from different locations in Chiang Mai, Thailand

Pathogen	Total no. of samples	No. of positive samples (%)	Viruses isolated from sampling locations:					
			Ang Kaew Reservoir^*a*^	Suandok Canal^*b*^	Sompech Canal^*b*^	Ping River^*c*^	Mae Kha Canal^*d*^	Buak Hard Reservoir^*a*^
Adenovirus	126	1 (0.8)	0	0	0	0	1	0
Astrovirus	126	6 (4.8)	0	0	0	0	6	0
Enterovirus	126	1 (0.8)	0	0	0	0	1	0
Human parechovirus	126	4 (3.2)	0	1	0	0	3	0
Rotavirus	126	1 (0.8)	0	0	0	1	0	0
Saffold virus	126	4 (3.2)	0	0	0	0	3	1
All targeted viruses	126	17 (13.5)	0	1	0	1	14	1

a Reservoir.

b Irrigation water.

c River water.

d Wastewater.

### Seasonality and diversity of enteric viruses detected in environmental water samples.

**FIG 2:**
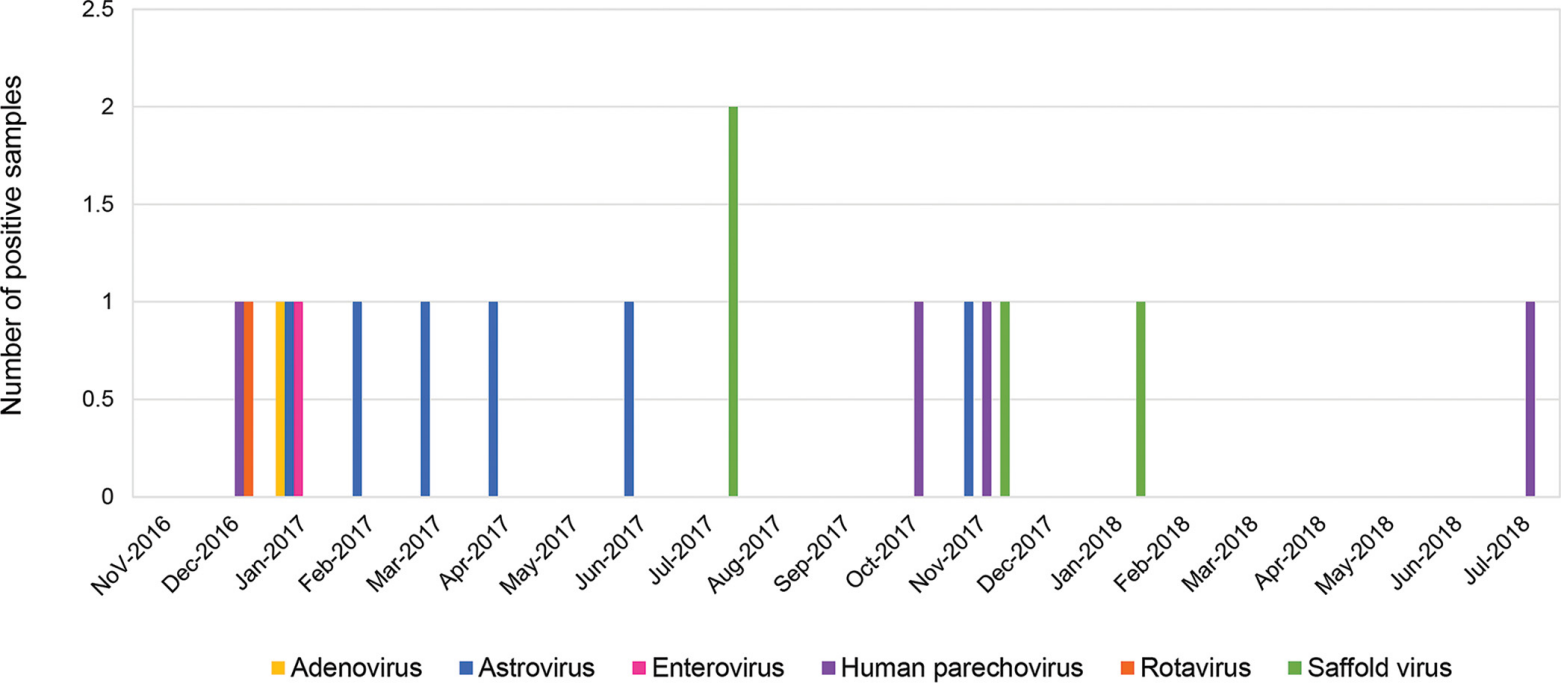
Monthly distribution of the six different enteric viruses investigated in this study.

Monthly distribution of enteric viruses was observed from November 2016 to July 2018 (Fig. 2). Six astrovirus strains were detected from January to April, in June, and in November 2017, all of these being novel astrovirus MLB1. Human strains of parechovirus were detected in December 2016, October and November 2017, and July 2018. Two human parechovirus strains were identified as HPeV1, while the other two strains were identified as HPeV5 and 6. The two saffold viruses detected in July were SAFV2; the other two, detected in November 2017 and January 2018, were SAFV3. Rotavirus G4P[8] was detected in December 2016, while adenovirus 41 and coxsackievirus A were detected in January 2017.

### Overall picture of the contamination of environmental water by enteric viruses in Chiang Mai, Thailand.

**FIG 3:**
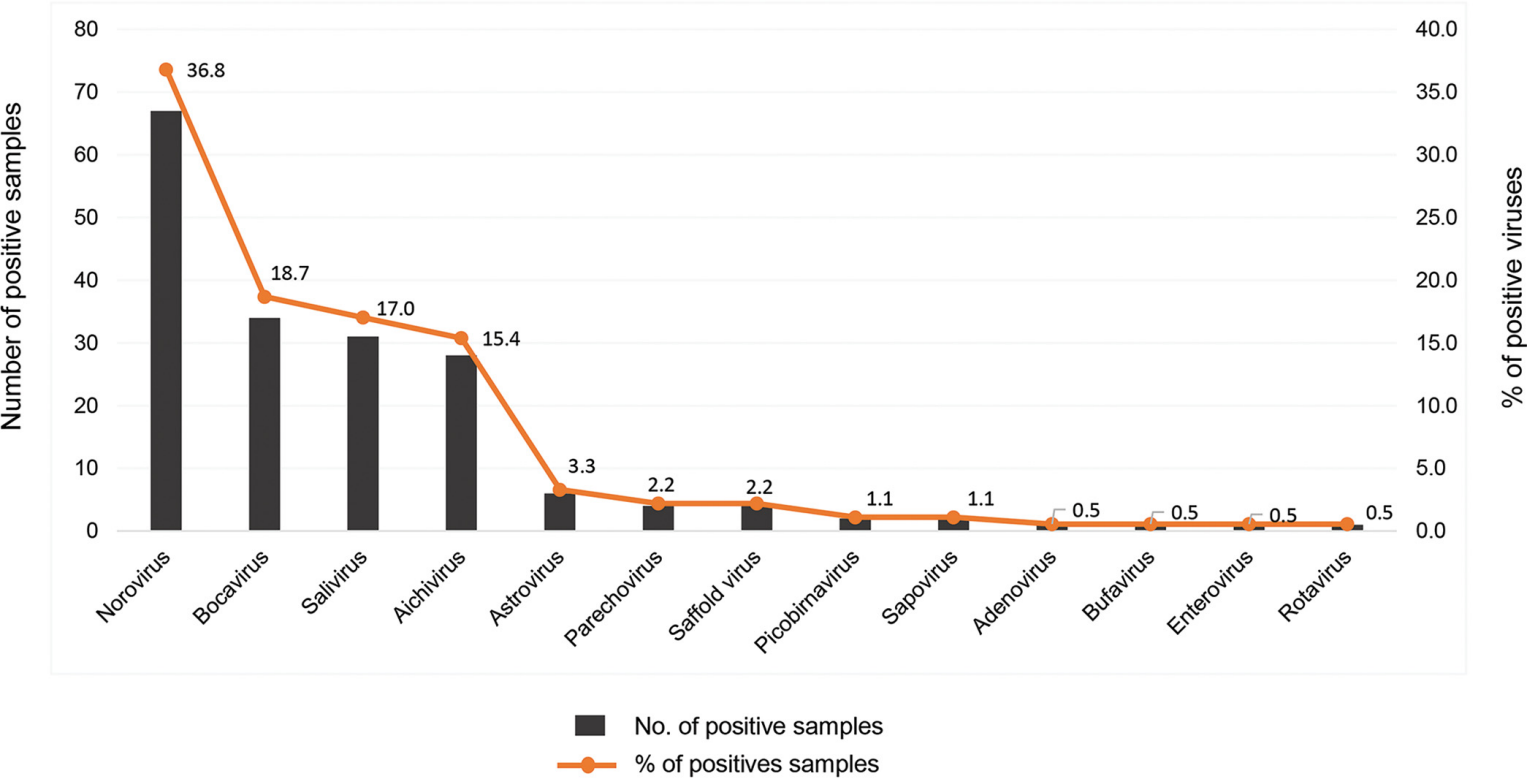
Enteric viruses (*n* = 13) in environmental water samples in Chiang Mai, Thailand. Adenovirus, astrovirus, enteroviruses, human parechovirus, rotavirus, and saffold virus were detected in the present study. Aichivirus, bufavirus, human bocavirus, noroviruses, sapovirus, picobirnavirus, and salivirus were detected in the same set of samples and reported previously (5–10). The water samples were concentrated using the PEG method and used for detecting each of the different viruses by PCR or RT-PCR.

**FIG 4:**
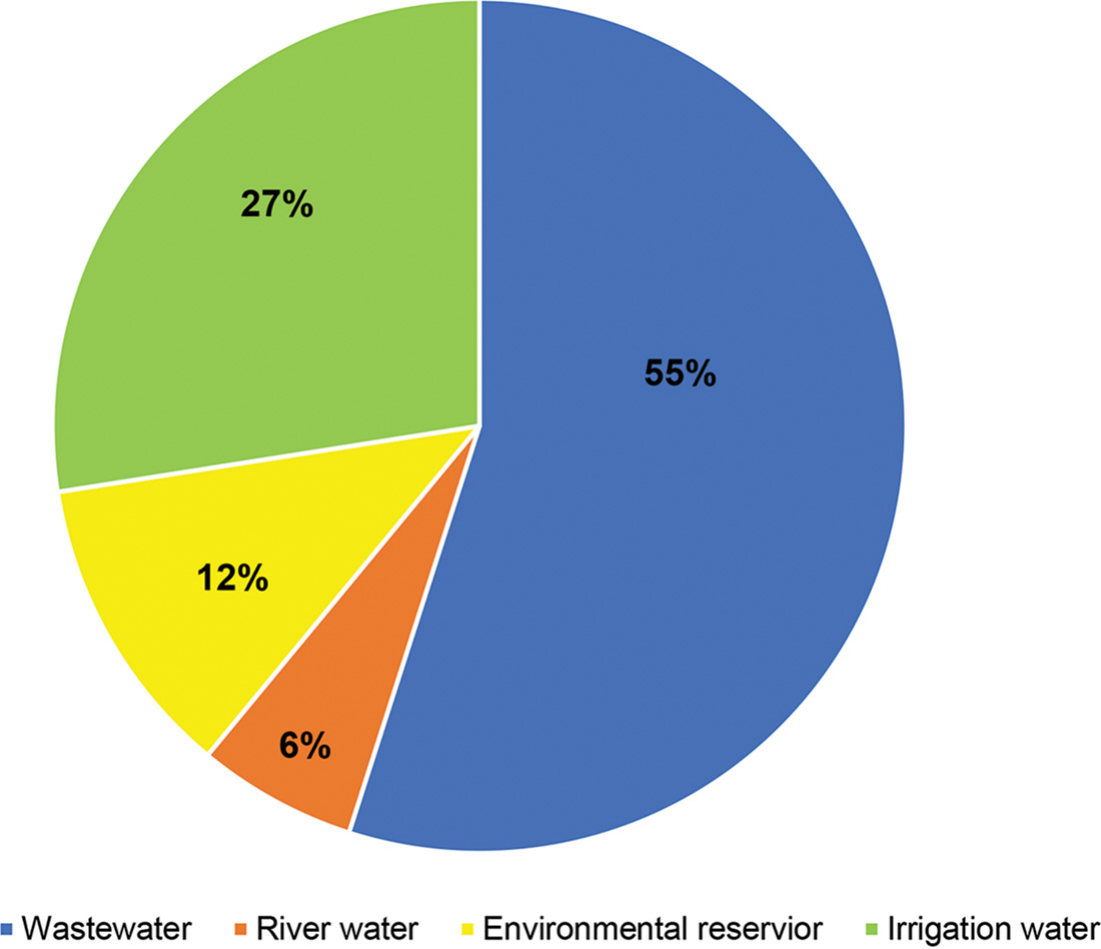
Prevalence of enteric viruses contaminating different types of environmental water. Notably, 182 strains of 13 different enteric viruses were detected.

In order to obtain an overall picture of enteric virus contamination in environmental water in the period 2016 to 2018 in Chiang Mai, Thailand, the data reported previously by our group from the same set of water samples were analyzed together with those obtained in this study, as shown in Fig. 3. Altogether, 13 human enteric viruses were detected in environmental water in Chiang Mai, Thailand. Of these, 6 enteric pathogens (adenovirus, astrovirus, enteroviruses, human parechovirus, rotavirus, saffold virus) were detected in the current study, and 7 (Aichivirus, bufavirus, human bocavirus, picobirnavirus, noroviruses and sapovirus, salivirus) were reported previously (5–10). From 126 water samples, after screening all 13 enteric viruses, 182 strains of viruses were detected. The prevalence of different enteric viruses was calculated from a total of the viruses detected (182 strains). The data showed that of a total of 182 enteric viruses detected, norovirus was the predominant enteric virus (67 strains, 36.8%), followed by human bocavirus (34 strains, 18.7%), salivirus (31 strains, 17.0%), Aichivirus (28 strains, 15.4%), astrovirus (6 strains, 3.3%), human parechovirus and saffold virus (4 strains each, 2.2%), picobirnavirus and sapovirus (2 strains each, 1.1%), and adenovirus, bufavirus, enteroviruses, and rotavirus (1 strain each, 0.5%). In addition, the prevalence of contamination by enteric viruses in different types of environmental water samples was also analyzed. Among the 182 virus strains detected in 126 water samples, wastewater and irrigation water were the first and second most polluted types of water, respectively. A total of 100 incidences (55%) of enteric virus strains were detected in wastewater samples, whereas 50 (27%) were detected in irrigation water samples (Fig. 4). In contrast, contamination by enteric viruses in environmental reservoir and river water was found to a lesser degree, at 21.1% and 11.6%, respectively. It should be noted that two or more enteric viruses were detected in each wastewater sample.

## DISCUSSION

Human enteric viruses are excreted in the feces of an infected person and are mainly transmitted through the fecal-oral route by the ingestion of contaminated food and/or water. Surveillance of virus contamination in environmental water and food materials is necessary in order to monitor and risk-assess waterborne virus-related human infection and public health impacts. The enteric viruses that are most often implicated in waterborne disease outbreaks include noroviruses, adenovirus, astrovirus, enteroviruses, and rotavirus (2). In addition to these viruses, additional viruses of less epidemiologic importance, such as human parechovirus and saffold virus, are also capable of waterborne transmission. This study demonstrated the presence of adenovirus, astrovirus, enteroviruses, human parechovirus, rotavirus, and saffold virus in environmental water samples at different rates of prevalence, ranging from 0.8 to 4.8%.

Adenovirus, astrovirus, enteroviruses, and rotavirus have been detected in water samples in several countries, with different prevalences ranging from 20 to 100% in China, 50 to 100% in Brazil, 23 to 46% in Canada, and 16.4 to 100% Pakistan (11–18). In contrast to common enteric viruses, studies of the surveillance of human parechovirus in water samples are very limited, with only one carried out in Japan being published reporting a prevalence of 18% (19). In addition, saffold virus in water samples has been recently reported by studies in Argentina, Iran, and Italy, with a prevalence of 5 to 47% (20–22). The present study is reporting, for the first time, the detection of adenovirus, astrovirus, enteroviruses, human parechovirus, and saffold virus in environmental water in Thailand. The levels of prevalence of enteric viruses detected in this study, which ranged from 0.8 to 4.8%, are relatively lower than those reported from other countries around the world. Several factors might contribute to this variation, including differences in the methodologies used for viral concentration, viral detection, and type and location of water sample collection. The detection method is one of the key factors contributing to the sensitivity of the detection rate. For example, real-time PCR is more sensitive than conventional PCR. The levels of prevalence of virus detection using real-time PCR reported by several studies (14, 15, 20, 22) were higher than the studies using conventional PCR (11, 13, 21), a phenomenon observed in our study as well. In addition, different primer sets targeting viruses of interest and different types of water samples used in the different studies may also affect the viral detection rate. The current study included four types of water sample, natural water reservoir, river water, irrigation water, and wastewater, while previous studies focused only on a particular type of water, such as tap water (11, 13), wastewater (12, 19), river water (22), or raw sewage (21, 23). Taken together, these factors could contribute to the differences in the rate of detection between one study and the others.

Our previous studies reported a wide range of gastroenteritis viruses in water samples, including Aichivirus, bufavirus, human bocavirus, noroviruses, picobiranvirus, salivirus, and sapovirus, with the prevalence ranging from 0.8 to 27.0% (5–10). Taking the data obtained from the present study together with those from our previous studies, 13 human enteric viruses have been found contaminating the environmental water in Chiang Mai, Thailand, during 2016 to 2018. Those with the highest prevalence were noroviruses, human bocavirus, salivirus, and Aichivirus at 22.2 to 27.0%. The remainder of the enteric viruses were detected at a much lower prevalences of 0.8 to 4.8%. The data indicate that a wide variety of human enteric viruses are circulating in environmental water, which could be a potential source of human infection. The finding highlights the need for adequate and proper control of the disposal and treatment of wastewater to reduce the spread of viral pathogens in environmental water.

Enteric viruses are commonly found in sewage and wastewater, and there may be various types of virus in an individual sample (2, 24). One of the limitations of the present study is that only major types of enteric viruses have been detected and identified using PCR, RT-PCR, and sequencing. In order to detect all virus strains contaminating the environmental water, more sensitive methods, such as real-time PCR or next generation sequencing, are required. Information derived from molecular detection of the viral genome may indicate contamination of the water samples by a virus; however, the level of infectivity of the detected virus needs to be determined to assess the risk of viral transmission to humans. In order to evaluate viral infectivity, infectious titer assays such as the plaque assay and focus forming assay need to be done. Enteric viruses have been reported to survive and maintain infectivity in environments such as seawater, freshwater, and soil for long periods of time, up to 130, 120, and 100 days, respectively (25). Several studies have demonstrated the relationship between viral persistence (as detected by molecular methods) and infectivity (as detected by cytopathic effect assay), indicating that degradation of viral nucleic acids shows a strong correlation with the loss of viral infectivity (26–28). Skraber et al. (27) reported that the loss of detection of the poliovirus genome is correlated to the disappearance of viral infectivity. The reports suggest that the occurrence of a viral genome may serve as an indicator for the presence of infectious viruses in the environment. Therefore, contamination of environmental water by viruses could be a potential source of human infection.

In conclusion, this study provides an enhanced view of enteric viruses that had not been of concern with regard to waterborne transmission in the past and may potentially be important waterborne pathogens. The data allow for a better understanding of viral dynamics and the epidemiologic importance of viral contamination in environmental water.

## MATERIALS AND METHODS

### Water sample collection and viral concentration.

A total of 126 water samples were collected monthly from six different sampling sites in Chiang Mai, a northern part of Thailand from November 2016 to July 2018. Four types of water samples were collected from 6 different locations: 2 reservoirs (Ang Kaew and Buak Hard Garden), 2 sites of irrigation water (the Suandok and Sompech Canals), river water (the Ping River), and wastewater (Mae Kha Canal) (Fig. 1). Overall, from 126 water samples, 42, 42, 21, and 21 water samples were from reservoirs, irrigation water, river water, and wastewater, respectively. Samples (100 mL) were collected, kept on ice in a transport box, and shipped to the laboratory on the same day. On arrival at the laboratory, samples were immediately concentrated using the polyethylene glycol (PEG) precipitation method (29) with minor modifications as described previously (6, 23). In brief, 8 g of PEG 6000 and 2.3 g of NaCl were added to 100 mL of water sample and mixed with a magnetic stirrer at 4°C overnight. The samples were then centrifuged at 10,000 × *g* at 4°C for 30 min. The pellet was resuspended in 200 μL of nuclease-free water in preparation for the next step.

### Viral genomic extraction and cDNA synthesis.

A total of 200 μL of concentrated water was used in the extraction of viral nucleic acid using a viral nucleic acid extraction kit (Geneaid, Taiwan) in accordance with the manufacturer’s instructions. A final volume of 50 μL of nucleic acid extract was obtained. Then, 10 μL of nucleic acid extract was used immediately for reverse transcription, and the remainder was stored at −80°C. The cDNA was synthesized from the viral RNA extract using the RevertAid first-strand cDNA synthesis kit (Thermo Scientific, USA) according to the manufacturer’s protocol. The cDNA was then used for RT-PCR, and the remainder of the cDNA was kept at −20°C.

### Detection of enteric viruses by PCR and RT-PCR.

Detection of adenovirus was performed by PCR, whereas detection of the other RNA viruses–astrovirus, enteroviruses, human parechovirus, rotavirus, and saffold virus—was carried out by RT-PCR using GoTaq DNA polymerase (Promega, USA) and the primers described in earlier studies (30–34) and shown in Table 2. The thermocycling conditions used for the detection of adenovirus, astrovirus virus, enterovirus, human parechovirus, and rotavirus consisted of 94°C for 3 min, 35 cycles of 94°C for 1 min, 50°C for 1 min, and 72°C for 1 min and then final extension at 72°C for 7 min. For saffold virus, the gradient cycles were the same as described above with the exception of the annealing temperatures used in the first and second rounds of amplification, which were performed at 55°C and 65°C, respectively. The amplified PCR products were analyzed by 1.5% agarose gel electrophoresis to visualize the PCR product size of each enteric virus. Positive and negative stool samples of each target virus were extracted from patients with acute gastroenteritis to confirm the virus genotype and were stored in the laboratory at −80°C. These were run through the same process of viral genome extraction, RT, and PCR to ensure that the entire process of virus detection was reliable and no contamination occurred throughout the process of viral detection.

**Table 2:** List of specific primers used for detection of six enteric viruses in this study^*a*^

Target virus	Primer	Polarity	Target region	Sequence (5′–3′)	PCR product size (bp)	Reference
Adenovirus	Ad1	+	Hexon	TTCCCCATGGCICAYAACAC	482	30
	Ad2	−	Hexon	CCCTGGTAKCCRATRTTGTA		
Astrovirus	SF0073	+	ORF1b	GATTGGACTCGATTTGATGG	409	32
	SF0076	−	ORF1b	CTGGCTTAACCCACATTCC		
Enterovirus	F1	+	5′UTR	CAAGCACTTCTGTTTCCCCGG	440	33
	R1	−	5′UTR	ATTGTCACCATAAGCAGCCA		
Human parechovirus	ev22(+)	+	5′UTR	CYCACACAGCCATCCTC	270	31
	ev22(−)	−	5′UTR	TRCGGGTACCTTCTGGG		
Rotavirus A	sBeg9	+	VP7	GGCTTTAAAAGAGAGAATTTC	395	30
	VP7-1’	−	VP7	ACTGATCCTGTTGGCCATCCTTT		
Saffold virus	CF188	+	5′UTR	CTAATCAGAGGAAAGTCAGCAT	824	34
	CR990	−	5′UTR	GACCACTTGGTTTGGAGAAGCT		
	CF204	+	5′UTR	CAGCATTTTCCGGCCCAGGCTAA	539	
	CR718	−	5′UTR	GCTATTGTGAGGTCGCTACAGCTGT		

a −, negative; +, positive; ORF, open reading frame; UTR, untranslated region.

### Nucleotide sequencing.

The positive PCR products were purified using a gel/PCR DNA fragment extraction kit (Geneaid, Taiwan) according to the manufacturer’s instructions. The purified PCR products were direct sequenced with the Sanger sequencing method using an automatic genetic analyzer (Applied Biosystems) provided by Apical Scientific Sdn. Bhd., Malaysia (formerly known as First BASE Laboratories Sdn. Bhd.). The nucleotide sequences of target viruses were compared with those of the reference sequences available in the NCBI GenBank database. For initial identification of the genotype of the viruses, the Basic Local Alignment Search Tool (BLAST) was used to compare a query sequence to a set of reference sequences of known virus genotypes. The genotype of the reference sequence that was most closely related to the query was considered to be the strain of the virus. In addition, genotypes of enteric viruses were further identified by sequence and phylogenetic analyses. The G and P genotypes of rotavirus were determined by RT-PCR using specific primers, sBeg9/End9 for the VP7 gene and Con3/Con2 for the partial VP4, and nucleotide sequencing (35, 36).

### Data availability.

The raw data from this study are available from the corresponding author upon reasonable request.

## References

[1] Desselberger U. 2017. Viral gastroenteritis. Medicine (Abingdon) 45:690–694. doi:10.1016/j.mpmed.2017.08.005. 32288581 PMC7108362

[2] Gibson KE. 2014. Viral pathogens in water: occurrence, public health impact, and available control strategies. Curr Opin Virol 4:50–57. doi:10.1016/j.coviro.2013.12.005. 24440908 PMC7185559

[3] Rzezutka A, Cook N. 2004. Survival of human enteric viruses in the environment and food. FEMS Microbiol Rev 28:441–453. doi:10.1016/j.femsre.2004.02.001. 15374660

[4] Desselberger U, Gray J. 2013. Viral gastroenteritis. Medicine (Abingdon) 41:700–704. doi:10.1016/j.mpmed.2013.09.009. 32288575 PMC7108395

[5] Kumthip K, Khamrin P, Yodmeeklin A, Ushijima H, Maneekarn N. 2021. Contamination of Human Bocavirus Genotypes 1, 2, 3, and 4 in Environmental Waters in Thailand. Microbiol Spectr 9:e0217821. doi:10.1128/spectrum.02178-21. 34937184 PMC8694147

[6] Kumthip K, Khamrin P, Yodmeeklin A, Maneekarn N. 2020. Prevalence and genetic characterization of Aichivirus in environmental waters in Thailand. Food Environ Virol 12:342–349. doi:10.1007/s12560-020-09445-x. 33044663

[7] Khamrin P, Kumthip K, Thongprachum A, Sirilert S, Malasao R, Okitsu S, Hayakawa S, Ushijima H, Maneekarn N. 2020. Genetic diversity of norovirus genogroup I, II, IV and sapovirus in environmental water in Thailand. J Infect Public Health 13:1481–1489. doi:10.1016/j.jiph.2020.05.004. 32493670

[8] Bell N, Khamrin P, Kumthip K, Rojjanadumrongkul K, Nantachit N, Maneekarn N. 2020. Molecular detection and characterization of picobirnavirus in environmental water in Thailand. Clin Lab 66. doi:10.7754/Clin.Lab.2019.191013. 32390390

[9] Nantachit N, Khamrin P, Kumthip K, Malasao R, Maneekarn N. 2019. Molecular surveillance and genetic analyses of bufavirus in environmental water in Thailand. Infect Genet Evol 75:104013. doi:10.1016/j.meegid.2019.104013. 31446136

[10] Badru S, Khamrin P, Kumthip K, Yodmeeklin A, Surajinda S, Supadej K, Sirilert S, Malasao R, Okitsu S, Ushijima H, Maneekarn N. 2018. Molecular detection and genetic characterization of Salivirus in environmental water in Thailand. Infect Genet Evol 65:352–356. doi:10.1016/j.meegid.2018.08.023. 30145388

[11] Rashid M, Khan MN, Jalbani N. 2021. Detection of human adenovirus, rotavirus, and enterovirus in tap water and their association with the overall quality of water in Karachi, Pakistan. Food Environ Virol 13:44–52. doi:10.1007/s12560-020-09448-8. 33180282

[12] Rmadi Y, Elargoubi A, Gonzalez-Sanz R, Mastouri M, Cabrerizo M, Aouni M. 2022. Molecular characterization of enterovirus detected in cerebrospinal fluid and wastewater samples in Monastir, Tunisia, 2014–2017. Virol J 19:45. doi:10.1186/s12985-022-01770-w. 35303921 PMC8932122

[13] Kluge M, Fleck JD, Soliman MC, Luz RB, Fabres RB, Comerlato J, Silva JVS, Staggemeier R, Vecchia AD, Capalonga R, Oliveira AB, Henzel A, Rigotto C, Spilki FR. 2014. Human adenovirus (HAdV), human enterovirus (hEV), and genogroup A rotavirus (GARV) in tap water in southern Brazil. J Water Health 12:526–532. doi:10.2166/wh.2014.202. 25252356

[14] Ye XY, Ming X, Zhang YL, Xiao WQ, Huang XN, Cao YG, Gu KD. 2012. Real-time PCR detection of enteric viruses in source water and treated drinking water in Wuhan, China. Curr Microbiol 65:244–253. doi:10.1007/s00284-012-0152-1. 22645016

[15] Pang X, Gao T, Qiu Y, Caffrey N, Popadynetz J, Younger J, Lee BE, Neumann N, Checkley S. 2021. The prevalence and levels of enteric viruses in groundwater of private wells in rural Alberta, Canada. Water Res 202:117425. doi:10.1016/j.watres.2021.117425. 34284123

[16] Cuevas-Ferrando E, Perez-Cataluña A, Falcó I, Randazzo W, Sánchez G. 2022. Monitoring human viral pathogens reveals potential hazard for treated wastewater discharge or reuse. Front Microbiol 13:836193. doi:10.3389/fmicb.2022.836193. 35464930 PMC9026171

[17] Lin X, Zou R, Liu Y, Ji F, Tao Z, Xu A. 2021. Continuous detection of norovirus and astrovirus in wastewater in a coastal city of China in 2014–2016. Lett Appl Microbiol 73:418–425. doi:10.1111/lam.13530. 34176155

[18] Tao Z, Lin X, Liu Y, Ji F, Wang S, Xiong P, Zhang L, Xu Q, Xu A, Cui N. 2022. Detection of multiple human astroviruses in sewage by next generation sequencing. Water Res 218:118523. doi:10.1016/j.watres.2022.118523. 35525029

[19] Abe M, Ueki Y, Miura T, Kimura S, Suzuki Y, Sugawara N, Masago Y, Omura T, Watanabe S. 2016. Detection of human parechoviruses in clinical and municipal wastewater samples in Miyagi, Japan, in 2012–2014. Jpn J Infect Dis 69:414–417. doi:10.7883/yoken.JJID.2015.551. 26902212

[20] Aminipour M, Ghaderi M, Harzandi N. 2020. First occurrence of saffold virus in sewage and river water samples in Karaj, Iran. Food Environ Virol 12:75–80. doi:10.1007/s12560-019-09415-y. 31729639

[21] Bonanno Ferraro G, Mancini P, Veneri C, Iaconelli M, Suffredini E, Brandtner D, La Rosa G. 2020. Evidence of saffold virus circulation in Italy provided through environmental surveillance. Lett Appl Microbiol 70:102–108. doi:10.1111/lam.13249. 31742735

[22] López GR, Martinez LM, Freyre L, Freire MC, Vladimirsky S, Rabossi A, Cisterna DM. 2021. Persistent detection of cosavirus and saffold cardiovirus in Riachuelo River, Argentina. Food Environ Virol 13:64–73. doi:10.1007/s12560-020-09451-z. 33165867

[23] Thongprachum A, Fujimoto T, Takanashi S, Saito H, Okitsu S, Shimizu H, Khamrin P, Maneekarn N, Hayakawa S, Ushijima H. 2018. Detection of nineteen enteric viruses in raw sewage in Japan. Infect Genet Evol 63:17–23. doi:10.1016/j.meegid.2018.05.006. 29753903

[24] La Rosa G, Fratini M, della Libera S, Iaconelli M, Muscillo M. 2012. Emerging and potentially emerging viruses in water environments. Ann Ist Super Sanita 48:397–406. doi:10.4415/ANN_12_04_07. 23247136

[25] Fong TT, Lipp EK. 2005. Enteric viruses of humans and animals in aquatic environments: health risks, detection, and potential water quality assessment tools. Microbiol Mol Biol Rev 69:357–371. doi:10.1128/MMBR.69.2.357-371.2005. 15944460 PMC1197419

[26] Wetz JJ, Lipp EK, Griffin DW, Lukasik J, Wait D, Sobsey MD, Scott TM, Rose JB. 2004. Presence, infectivity, and stability of enteric viruses in seawater: relationship to marine water quality in the Florida Keys. Mar Pollut Bull 48:698–704. doi:10.1016/j.marpolbul.2003.09.008. 15041426

[27] Skraber S, Gassilloud B, Schwartzbrod L, Gantzer C. 2004. Survival of infectious Poliovirus-1 in river water compared to the persistence of somatic coliphages, thermotolerant coliforms and Poliovirus-1 genome. Water Res 38:2927–2933. doi:10.1016/j.watres.2004.03.041. 15223287

[28] Dubois E, Le Guyader F, Haugarreau L, Kopecka H, Cormier M, Pommepuy M. 1997. Molecular epidemiological survey of rotaviruses in sewage by reverse transcriptase seminested PCR and restriction fragment length polymorphism assay. Appl Environ Microbiol 63:1794–1800. doi:10.1128/aem.63.5.1794-1800.1997. 9143113 PMC168473

[29] Lewis GD, Metcalf TG. 1988. Polyethylene glycol precipitation for recovery of pathogenic viruses, including hepatitis A virus and human rotavirus, from oyster, water, and sediment samples. Appl Environ Microbiol 54:1983–1988. doi:10.1128/aem.54.8.1983-1988.1988. 2845860 PMC202790

[30] Yan H, Nguyen TA, Phan TG, Okitsu S, Li Y, Ushijima H. 2004. Development of RT-multiplex PCR assay for detection of adenovirus and group A and C rotaviruses in diarrheal fecal specimens from children in China. Kansenshogaku Zasshi 78:699–709. doi:10.11150/kansenshogakuzasshi1970.78.699. 15478645

[31] Joki-Korpela P, Hyypiä T. 1998. Diagnosis and epidemiology of echovirus 22 infections. Clin Infect Dis 27:129–136. doi:10.1086/514615. 9675466

[32] Finkbeiner SR, Holtz LR, Jiang Y, Rajendran P, Franz CJ, Zhao G, Kang G, Wang D. 2009. Human stool contains a previously unrecognized diversity of novel astroviruses. Virol J 6:161. doi:10.1186/1743-422X-6-161. 19814825 PMC2765957

[33] Zoll GJ, Melchers WJ, Kopecka H, Jambroes G, van der Poel HJ, Galama JM. 1992. General primer-mediated polymerase chain reaction for detection of enteroviruses: application for diagnostic routine and persistent infections. J Clin Microbiol 30:160–165. doi:10.1128/jcm.30.1.160-165.1992. 1370845 PMC265013

[34] Drexler JF, Luna LKDS, Stöcker A, Almeida PS, Ribeiro TCM, Petersen N, Herzog P, Pedroso C, Huppertz HI, Ribeiro HdC, Baumgarte S, Drosten C. 2008. Circulation of 3 lineages of a novel Saffold cardiovirus in humans. Emerg Infect Dis 14:1398–1405. doi:10.3201/eid1409.080570. 18760006 PMC2603095

[35] Gentsch JR, Glass RI, Woods P, Gouvea V, Gorziglia M, Flores J, Das BK, Bhan MK. 1992. Identification of group A rotavirus gene 4 types by polymerase chain reaction. J Clin Microbiol 30:1365–1373. doi:10.1128/jcm.30.6.1365-1373.1992. 1320625 PMC265294

[36] Gouvea V, Glass RI, Woods P, Taniguchi K, Clark HF, Forrester B, Fang ZY. 1990. Polymerase chain reaction amplification and typing of rotavirus nucleic acid from stool specimens. J Clin Microbiol 28:276–282. doi:10.1128/jcm.28.2.276-282.1990. 2155916 PMC269590

